# Manual of Histology

**Published:** 1882-01

**Authors:** 


### REVIEWS.

---

Manual of Histology. Edited and prepared by Thomas E. Satterthwaite, M.D., in association with Drs. Thomas Dwight, J. Collins Warren, Wm. F. Whitney, Clarence J. Blake, and C. H. Williams, of Boston; Dr. J. H. C. Simes, of Philadelphia; Dr. B. F. Westbrook, of Brooklyn; and Drs. E. C. Wendt, A. Mayer, R. W. Amidon, A. R. Robinson, W. R. Birdsall, D. B. Delavan, C. L. Dana, and W. H. Porter, of New York City. New York: William Wood & Co. 1881.

This practical treat se on a fundamental branch of medicine, the importance of which is becoming daily more and more recognized, reflects the highest credit upon the able editor and the large staff of collaborators. The distinguishing features of the work are the eminently practical manner in which the various topics relating to the minute structure of the body are dealt with. It would appear that the editor and most of the contributors are, and have been, engaged in teaching Histology to different classes of students. This has clearly enabled them to learn exactly what the student needs in order to become familiar with the microscopical appearance and complexion of the body. The fact that Comparative Histology is duly recognized throughout the body, makes it of additional value to the veterinarian and student of comparative medicine. We know of no better work on this subject in the English language, and we feel great pleasure in welcoming its appearance as a timely contribution from American Histologists to the fascinating study of minute anatomy.

